# Whole Exome Sequencing in a Random Sample of North American Women with Leiomyomas Identifies *MED12* Mutations in Majority of Uterine Leiomyomas

**DOI:** 10.1371/journal.pone.0033251

**Published:** 2012-03-12

**Authors:** Megan M. McGuire, Alexander Yatsenko, Lori Hoffner, Mirka Jones, Urvashi Surti, Aleksandar Rajkovic

**Affiliations:** 1 Department of Obstetrics, Gynecology and Reproductive Sciences, Magee-Womens Research Institute, University of Pittsburgh, Pittsburgh, Pennsylvania, United States of America; 2 Department of Gynecologic Pathology, Magee-Womens Hospital of UPMC, Pittsburgh, Pennsylvania, United States of America; Tel Aviv University, Israel

## Abstract

Uterine leiomyomas (uterine fibroids) arise from smooth muscle tissue in the majority of women by age 45. It is common for these clonal tumors to develop from multiple locations within the uterus, leading to a variety of symptoms such as pelvic pain, abnormal uterine bleeding, and infertility. We performed whole exome sequencing on genomic DNA from five pairs of leiomyomas and corresponding normal myometrium to determine genetic variations unique to leiomyomas. Whole exome sequencing revealed that the gene encoding transcription factor MED12 (Mediator complex subunit 12) harbored heterozygous missense mutations caused by single nucleotide variants in highly conserved codon 44 of exon 2 in two of five leiomyomas. Sanger re-sequencing of *MED12* among these five leiomyomas confirmed the two single nucleotide variants and detected a 42 base-pair deletion within exon 2 of *MED12* in a third leiomyoma. *MED12* was sequenced in an additional 143 leiomyomas and 73 normal myometrial tissues. Overall, *MED12* was mutated in 100/148 (67%) of the genotyped leiomyomas: 79/148 (53%) leiomyomas exhibited heterozygous missense single nucleotide variants, 17/148 (11%) leiomyomas exhibited heterozygous in-frame deletions/insertion-deletions, 2/148 (1%) leiomyomas exhibited intronic heterozygous single nucleotide variants affecting splicing, and 2/148 (1%) leiomyomas exhibited heterozygous deletions/insertion-deletions spanning the intron 1-exon 2 boundary which affected the splice acceptor site. Mutations were not detected in *MED12* in normal myometrial tissue. *MED12* mutations were equally distributed among karyotypically normal and abnormal uterine leiomyomas and were identified in leiomyomas from both black and white American women. Our studies show an association between *MED12* mutations and leiomyomas in ethnically and racially diverse American women.

## Introduction

Uterine leiomyomas, better known as fibroid tumors, are clinically apparent in nearly 25% of women by age 45, and they cause major morbidity in American women. More than 200,000 surgeries are performed each year to either remove the leiomyomatous tumors (myomectomy) or the entire uterus (hysterectomy) [1], [2]. These tumors are responsive to steroid hormones such as estrogen and progesterone, and they often shrink following menopause [3]. Approximately half of all leiomyomas are asymptomatic, while the rest cause pelvic pressure and pain, menometrorrhagia, anemia, premature labor and infertility. These symptoms are intensified by the common occurrence of multiple tumors within a single uterus, often necessitating surgical intervention [4].

Previous genetic analyses of leiomyomas have established that approximately one half of these tumors have an abnormal karyotype, while the other half are karyotypically normal. We recently showed that a subset of karyotypically normal leiomyomas contained microdeletions and microduplications, but there is still a large proportion of karyotypically normal leiomyomas that have no known lesions [5]. In karyotypically abnormal leiomyomas, cytogenetic aberrations commonly include deletions in 7q, trisomy of chromosome 12, and various translocations between chromosomes 12 and 14 involving the *high mobility group AT-hook 2* (*HMGA2*) gene at 12q15, which encodes a transcriptional regulator [6], [7], [8], [9]. Cytogenetic abnormalities are likely a reflection of general genomic instability as is true for other tumors. Mutations in leiomyomas that cause such genomic instabilities are unknown. Furthermore, heterozygous germline mutations in *fumarate hydratase* (*FH*) can cause a rare disorder of hereditary leiomyomatosis and renal cell carcinoma (HLRCC [MIM 150800]) [10], [11]. However, fumarate hydratase does not appear to play an important role in the non-syndromic forms of uterine leiomyomas [12].

## Methods

### Patient information

This study was approved by the Institutional Review Board of the University of Pittsburgh as an exempt study (Pittsburgh, PA; IRB# PRO11120169), and informed consent was not obtained. All leiomyoma and myometrial samples were derived from specimens that were originally obtained for clinical treatment or pathology purposes only, and specimens did not contain any personal identifiers or linkage codes. Samples were collected from women who were diagnosed with uterine leiomyomas and underwent medically indicated abdominal hysterectomy. Chromosome analysis was performed using standard G-banding technique. Whole exome sequencing on the initial five pairs of samples from five different individuals was performed on randomly selected, karyotypically normal leiomyomas. Subsequent DNA sequencing was performed on 143 randomly selected leiomyomas from our biobank of more than 500 samples. The histology of all samples was reviewed by a board certified gynecologic pathologist.

### Nucleic acid extraction and preparation

Genomic DNAs from all leiomyomas and corresponding myometrial samples were extracted from 100 mg of freshly frozen tissue using the Gentra Puregene Blood Kit (QIAGEN, Valencia, CA, USA), according to the manufacturer's protocol. RNA was extracted from a randomly selected group of leiomyoma samples which harbored *MED12* mutations. Tissue samples stored at −80°C were placed in chilled RNA*later*®-ICE (Invitrogen, Carlsbad, CA, USA) for at least 16 hrs at −20°C to allow for tissue thawing under preservative conditions prior to RNA isolation. TRIzol® Reagent (Invitrogen, Carlsbad, CA, USA) was used to carry out RNA isolations according to the manufacturer's protocol. RNA samples were converted to cDNA using SuperScript® III First-Strand Synthesis System (Invitrogen, Carlsbad, CA, USA), according to the manufacturer's protocol.

### Whole exome capture and DNA sequencing

Ten genomic DNA samples (five leiomyomas and five corresponding myometrial samples) were subjected to in-solution exome enrichment via the SureSelect Human All Exon Kit v2 (Agilent, Santa Clara, CA). Following exome capture, the samples were submitted for single read high-throughput sequencing on the Genome Analyzer IIx (Illumina Inc., San Diego, CA, USA).

### Sequencing data analysis

Exome sequencing raw data files were received in FASTQ format and converted to FASTA format using default settings for NextGENe v2.16 software (SoftGenetics, State College, PA, USA). NextGENe was also used for sequence alignment against reference human genome assembly GRCh37/hg19. Minimum coverage of 5 sequencing reads per base-pair (bp) was required for variant calling and variants in less than 20% of the sequencing reads were considered sequencing artifacts. Nucleotides in exons and within 10 bps of the exon-intron junctions were the focus of further variant analysis.

### Variant filtering

The initial steps of variant filtering for the exome sequencing data were carried out using NextGENe software; later filtering steps were performed on exported NextGENe files using the functions available in a spreadsheet. Each pair of leiomyoma and corresponding normal myometrial tissue was compared independently. Our focus was limited to DNA variants which were present in the leiomyoma but absent in the corresponding normal myometrium. Minimum coverage of 20 sequencing reads per bp was required for this stage of variant filtering. Due to the presence of common variants in both samples, we required the frequency of the reference allele in the leiomyoma sample to be at least 25% less than the frequency of the reference allele in the corresponding myometrial sample in order for the variant to pass the first filtering step. Variants outside of the exons and exon-intron junctions (10 bps flanking each exon) were excluded from further analysis. The variant lists for each tissue type (leiomyoma/myometrium) were compared against each other to elucidate variants which were unique to the leiomyoma. DNA variants present in the Single Nucleotide Polymorphism database v132 (dbSNP132) were removed (http://www.ncbi.nlm.nih.gov/projects/SNP/). Synonymous single nucleotide variants (SNVs) were also removed. Finally, DNA variants were evaluated with protein prediction tools, PolyPhen-2 and SIFT (http://genetics.bwh.harvard.edu/pph2/; http://sift.jcvi.org/), to reveal variants which were predicted to have an impact on protein function. The final variant lists were compared across samples to identify genes commonly mutated in multiple leiomyomas as a means to focus further study.

### *MED12* variant validation

Exome sequence data analysis identified one nucleotide in codon 44 of exon 2 of *MED12* which was uniquely mutated in two of five leiomyomas. To validate the presence of the SNVs, the five pairs of leiomyoma and corresponding myometrial samples underwent Sanger re-sequencing of *MED12*. Oligonucleotide primers were designed against *MED12* (NCBI Gene ID: 9968; chrX:70,338,406–70,362,304 [GRCh37/hg19]) using Primer3 (http:/frodo.wi.mit.edu/primer3/). Genomic DNA was amplified with *amfisure* PCR Master Mix (GenDEPOT, Barker, TX, USA) under the following conditions: 94°C for 3 min; 35 cycles of 94°C for 30 sec, 60°C for 30 sec, 72°C for 30 sec; and 72°C for 2 min. DNA sequences were evaluated using Sequencher software (Gene Codes Corp., Ann Arbor, MI, USA). Upon confirmation of the SNVs in two leiomyomas and identification of a deletion missed by whole exome sequencing (due to length) in a third leiomyoma, an additional 143 leiomyomas, 73 of which had corresponding myometrial samples, were screened for variants in *MED12* via Sanger sequencing, as described above. *MED12* variant data was deposited in the Single Nucleotide Polymorphism database (dbSNP; http://www.ncbi.nlm.nih.gov/projects/SNP/).

### cDNA sequencing

*MED12* cDNAs of exon 2 and flanking regions (exons 1 and 3) in ten randomly selected leiomyomas with *MED12* variants were sequenced to verify that the mutated allele was actively expressed in each leiomyoma. Oligonucleotide primers were designed using Primer3. cDNA was amplified with *amfisure* PCR Master Mix under the previously specified conditions. PCR products were gel purified using the QIAGEN Gel Extraction Kit (QIAGEN, Valencia, CA, USA) prior to Sanger sequencing.

### Statistical analysis

A one-tailed Fisher's exact test was performed to determine statistical significance of associations between categorical variables, and a *p* value<0.05 was considered to be significant.

## Results

To search for gene(s) that associate with leiomyomas, we examined the genomic DNA of leiomyomas and corresponding myometrial samples from five individuals via whole exome enrichment and high-throughput DNA sequencing. We analyzed DNA variants in exons and within 10 base-pairs (bps) of exon-intron junctions, excluding single nucleotide variants (SNVs) that populate the Single Nucleotide Polymorphism database v132 (dbSNP132), as well as those that did not affect the protein sequence. Among analyzed DNA variants, 24 were predicted to be damaging at the protein level (Table 1). We hypothesized that potentially damaging mutations within the same gene would be present in multiple leiomyomas. *MED12* (*Mediator complex subunit 12*; Xq13.1) was the only gene that presented with damaging variants in more than one leiomyoma. Two novel heterozygous single nucleotide variants (SNVs) were identified at the same position in exon 2 of *MED12* (codon 44) in two of the five leiomyomas (c.130G>A, p.G44S; c.130G>C, p.G44C), but not in the corresponding myometrial samples. Both SNVs were predicted to be damaging at the protein level. Sanger re-sequencing confirmed the presence of the two heterozygous *MED12* SNVs identified by whole exome sequencing and also detected a heterozygous 42 bp deletion predicted to delete fourteen amino acids in exon 2, including codon 44 (c.122_164del42; p.V41_D54del), in a third leiomyoma. The 42 bp deletion was not identified during exome data analysis due to the size of the deletion, as the average length of each sequencing read generated by exome sequencing was 74 bps. Overall, three of the five leiomyomas (60%) expressed one mutant *MED12* allele, and no variants were detected in *MED12* in the matched normal myometrial samples.

**Table 1:** Whole exome data filtering schema for DNA variants.

Sample ID	Variants in exons and exon-intron junctions^a^	Variants unique to leiomyoma^b^	Filtered variants^c^	Filtered SNVs		Filtered dels/indels		Damagingvariants^h^
				*Exonic* d	*Intronic* e	*Exonic* f	*Intronic* g	
L1^i^	13,654	355	13	9	-	2	2	5
L2	13,477	456	18	14^i^	1	2	1	5^i^
L3	13,976	342	18	12^i^	1	4	1	3^i^
L4	12,799	387	8	6	-	1	1	2
L5	13,513	373	18	11	2	5	-	9

Genomic DNAs from five pairs of leiomyomas and corresponding myometrial samples underwent whole exome enrichment and sequencing. After sequence alignment, each pair of leiomyoma and corresponding myometrium was independently analyzed using NextGENe software (represented by each row in the table; SoftGenetics, State College, PA). The DNA variants in each tissue (leiomyoma/normal myometrium) were compared to reveal mutations which were unique to the leiomyoma. After filtering against the Single Nucleotide Polymorphism database v132 (dbSNP132; http://www.ncbi.nlm.nih.gov/projects/SNP/) and applying protein prediction software tools, PolyPhen-2 and SIFT (http://genetics.bwh.harvard.edu/pph2/; http://sift.jcvi.org/), we identified *MED12* as the only gene commonly mutated in two or more leiomyomas. The minimum base coverage threshold was 20 sequencing reads per base-pair for variant filtering.

a All DNA variants in exons and within 10 base-pairs (bps) of exon-intron junctions in introns.

b DNA variants unique to the leiomyoma in exons and within 10 bps of exon-intron junctions in introns.

c DNA variants unique to the leiomyoma in exons and within 10 bps of exon-intron junctions in introns which were not found in dbSNP132.

d Exonic single nucleotide variants (SNVs) unique to the leiomyoma which caused a change in the protein sequence and were not found in dbSNP132.

e SNVs unique to the leiomyoma located in introns within 10 bps of exon-intron junctions which were not found in dbSNP132.

f Exonic deletions/insertion-deletions (dels/indels) unique to the leiomyoma which were not found in dbSNP132.

g Dels/indels unique to the leiomyoma located in introns within 10 bps of exon-intron junctions which were not found in dbSNP132.

h DNA variants unique to the leiomyoma in exons and within 10 bps of exon-intron junctions in introns which were predicted to be damaging to the protein, not found in dbSNP132.

i Leiomyomas L2 and L3 harbored missense SNVs in exon 2 of *MED12* which were detected by exome sequencing and predicted to be damaging. L1 contained a 42 bp deletion in exon 2 of *MED12* which was undetected by exome sequencing but revealed via Sanger sequencing.

**Figure 1:**
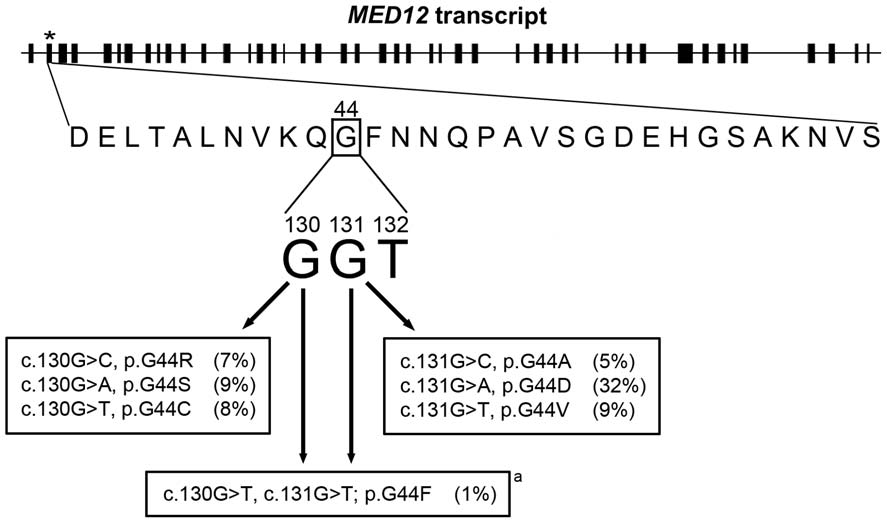
Variants in codon 44 in exon 2 of *MED12* accounted for 71% of mutations in uterine leiomyomas. This schema depicts the full-length human *MED12* transcript which contains 45 coding exons (6,531 base-pairs/2,177 amino acids). Exon 2, denoted by the asterisk, is located near the N terminus and contains 105 base-pairs/35 amino acids. Codon 44, which encodes glycine (boxed G), harbored single nucleotide variants in 71/100 (71%) mutated leiomyomas. These variants replaced guanines at nucleotide positions 130 and 131. All variants changed the amino acid encoded by codon 44, and these variants were predicted to be damaging. The percentage of the total number of mutated leiomyomas (100) harboring each variant is noted in parentheses. **^a^** One leiomyoma exhibited two consecutive single nucleotide variants at positions 130 and 131.

Whole exome sequencing yielded *MED12* as a candidate gene associated with uterine leiomyomas. We then sequenced *MED12* in an additional 143 leiomyomas and 73 normal myometrial samples; the leiomyomas were all derived from different individuals and the corresponding myometrial samples were taken from 73 of those individuals. *MED12* variants were not detected in the 73 normal myometrial samples; conversely, one mutant allele was detected in 97/143 leiomyomas (67.8%; Table 2). We observed 76 heterozygous missense SNVs, 68 of which occurred at positions 130 and 131 in codon 44 (68/76; 89.5%) and caused the following amino acid changes: G44R; G44S, G44C, G44A, G44D, and G44V (Figure 1). Additionally, two other heterozygous missense SNVs were detected – c.107T>G; L36R (3/143; 2.1%) and c.128A>C; Q43P (5/143; 3.5%). One leiomyoma exhibited two consecutive heterozygous SNVs in codon 44 (c.130G>T and c.131G>T; G44F). All of the amino acid modifications were predicted to be damaging by PolyPhen-2 and SIFT. Eighteen tumors (18/143; 12.6%) exhibited heterozygous in-frame deletions/insertion-deletions, ranging in size from two to fifteen amino acids. Two of the eighteen deletions/insertion-deletions spanned the intron 1-exon 2 junction, causing a predicted loss of the splice acceptor, in addition to other amino acids. Furthermore, one SNV located near the intron 1-exon 2 junction caused an insertion of two amino acids between codons 33 and 34 in two leiomyomas. We therefore observed a total of 100/148 (67.6%) leiomyomas with *MED12* variants, while no variants were detected in *MED12* in the normal myometrial samples (*p*<0.0001). These results suggest a strong association between somatically acquired variants in *MED12* and leiomyomata.

**Table 2:** DNA variants in leiomyomas were confined to exon 2 of *MED12*.

Variant location^a^	Variant type^b^	Nucleotide change	Protein change	Mutated leiomyomas^c^
Splice site	SNV	IVS1-8T>A	p.E33_D34insPQ	2 (1.4%/2.0%)
	Deletion/Indel	IVS1-1_139del41	Splice acceptor loss	1 (0.7%/1.0%)
		IVS1-2_141del44insAG	Splice acceptor loss	1 (0.7%/1.0%)
Exon 2	SNV	c.107T>G	p.L36R	3 (2.0%/3.0%)
		c.128A>C	p.Q43P	5 (3.4%/5.0%)
		c.130G>C	p.G44R	7 (4.7%/7.0%)
		c.130G>A	p.G44S	9 (6.1%/9.0%)
		c.130G>T	p.G44C	8 (5.4%/8.0%)
		c.131G>C	p.G44A	5 (3.4%/5.0%)
		c.131G>A	p.G44D	32 (21.6%/32.0%)
		c.131G>T	p.G44V	9 (6.1%/9.0%)
		c.130G>T;c.131G>T	p.G44F	1 (0.7%/1.0%)
	Deletion/Indel	c.103_138del36	p.E35_N46del	1 (0.7%/1.0%)
		c.107_111del5insGC	p.L36_T37delinsR	1 (0.7%/1.0%)
		c.111_155del45	p.A38_S52del	1 (0.7%/1.0%)
		c.113_121del9	p.A38_N40del	1 (0.7%/1.0%)
		c.117_122del6	p.N40_V41del	1 (0.7%/1.0%)
		c.118_132del15	p.N40_G44del	1 (0.7%/1.0%)
		c.118_134del17insTA	p.N40_F45delinsY	1 (0.7%/1.0%)
		c.118_146del29insTT	p.N40_P49delinsF	1 (0.7%/1.0%)
		c.122_148del27	p.V41_P49del	1 (0.7%/1.0%)
		c.122_163del42	p.V41_D54del	1 (0.7%/1.0%)
		c.123_152del30	p.K42_V51del	1 (0.7%/1.0%)
		c.126_131del6	p.K42_G44delinsN	1 (0.7%/1.0%)
		c.126_140del15	p.K42_F45del	1 (0.7%/1.0%)
		c.129_137del9	p.Q43_N46delinsH	1 (0.7%/1.0%)
		c.129_143del15	p.G44_Q48del	1 (0.7%/1.0%)
		c.133_144del12	p.F45_Q48del	1 (0.7%/1.0%)
		c.149_163del15	p.A50_D54del	1 (0.7%/1.0%)

a Splice site variants were located in intron 1 within 10 base-pairs of the intron 1-exon 2 junction.

b Variants were classified as a single nucleotide variant (SNV) or a deletion/insertion-deletion (indel).

c The number of leiomyomas with each specific variant is followed in parentheses by the percentage of the total number of leiomyomas under study (148) and the percentage of the total number of mutated leiomyomas (100), respectively.

All of the *MED12* variants that we observed in uterine leiomyomas were heterozygous. Since random X chromosome inactivation allows either the wild-type or the mutant allele to be expressed, we examined whether mutated *MED12* transcripts were expressed in leiomyomas harboring *MED12* DNA variants [13]. We isolated RNA and generated cDNA from a random selection of ten leiomyomas known to carry *MED12* DNA variants. Sanger sequencing of cDNAs from these leiomyomas revealed that all of the transcripts derived solely from the mutant *MED12* alleles. These results are consistent with the interpretation that leiomyomatous tumors with *MED12* DNA variants express a mutant form of the *MED12* protein.

Karyotype data was available for 134 leiomyomas: 85 (63%) were karyotypically normal, while 49 (37%) had various cytogenetic abnormalities (Table S1). The proportions of karyotypically normal and abnormal leiomyomas were consistent with previous studies [14], [15]. Fifty-nine of 85 leiomyomas (69%) with a normal karyotype harbored *MED12* variants, while 31/49 leiomyomas (63%) with an abnormal karyotype had *MED12* variants. These results were not statistically significant (*p* = 0.29). We also had racial information on 143 leiomyomas. Leiomyomas under analysis were obtained from 23 black American women and 120 white American women. The frequency of *MED12* mutation was 78% (18/23) in black American women and 66% (79/120) in white American women, but the association between race and *MED12* status was not statistically significant (*p* = 0.36). The frequency of *MED12* mutation was higher when multiple tumors were detected in a single uterus (72.5%; 87/120) as opposed to a single tumor (41%; 9/22) (*p*<0.01).

## Discussion

Uterine leiomyomas are benign smooth muscle tumors that can emanate from anywhere in the uterus and distort uterine anatomy and function. Leiomyomas are the leading cause of dysfunctional uterine bleeding and hysterectomies. The clonal origin of leiomyomas makes them genetically homogenous. However, karyotypes between individual leiomyomas differ, and abnormal karyotypes are identified in approximately 40% of cases [14], [15], [16]. Rare hereditary leiomyomatosis and renal cell carcinoma syndrome (HLRCC [MIM 150800]) is caused by heterozygous germline mutations in the gene encoding fumarate hydratase, but the role(s) of individual genes in common leiomyomas is not well understood [10], [11]. The presence of *MED12* variants in nearly 70% of leiomyomas suggests that *MED12* plays an important role in the genesis of these tumors in both white and black American women.

*MED12*, *mediator complex subunit 12*, encodes one of at least 26 proteins that comprise the Mediator complex. This Mediator forms a molecular bridge between gene-specific transcription factors and RNA polymerase II (RNAP II) [17]. The Mediator is divided into four functional units: the head which binds RNAP II, the middle, the CDK subcomplex which exhibits histone kinase activity, and the tail which binds gene-specific enhancers. MED12 is part of the CDK subcomplex along with three other proteins: Cyclin C, CDK8 (Cyclin-dependent kinase 8), and MED13 (Mediator complex subunit 13). MED12 is necessary for CDK8 activation [18]; through this interaction, MED12 indirectly aids in transcriptional regulation, as CDK8/Cyclin C is capable of phosphorylating the carboxy-terminal domain (CTD) of RNAPII leading to subsequent transcription of genes with certain types of promoters [19]. CDK8 is a colorectal cancer oncogene and likely plays a role in tumorigenesis [20]. MED12 is also important in chromatin modification and transcriptional repression, independent of its role in CDK8 activation [21]. If the current association is causative, *MED12* is a tumor suppressor gene, leading to abnormal leiomyomatous growth when mutated.

The variants described here are not germline mutations; rather, they are solely restricted to the tumor. Since *MED12* is located on the X chromosome, random X chromosome inactivation results in sole expression of the mutated allele with consequent tumorigenesis. Interestingly, there was no association between karyotype and *MED12* mutation, in our study. Numerous karyotype abnormalities associate with leiomyomas, and it is likely that such chromosomal imbalances are a secondary consequence of mutations that derail normal cell cycle controls. We hypothesize that *MED12* variants deregulate the cell cycle in the normal myometrium, resulting in abnormal growth and genomic instability.

Germline *MED12* mutations are responsible for at least two forms of X-linked mental retardation: Opitz-Kaveggia syndrome (FG syndrome [MIM 305450]) and Lujan-Fryns syndrome (MIM 309520). The SNVs implicated in these syndromes are located in exons 21 and 22, affecting the carboxy-terminus of MED12 (p.R961W and p.N1000S, respectively) [22], [23]. These two syndromes are not associated with tumorigenesis and carrier females are reported to be “normal”. None of the syndromic mutations were present in the leiomyomas under study. The concentration of variants within exon 2 of *MED12* in a significant subset of leiomyomas indicates that this region of the protein may be involved in cell cycle control and tumor repression.

*MED12* mutations were recently described in leiomyomas from a group of Finnish women [24]. In Finnish women, *MED12* was found to be mutated in 70% (159/225) of tumors from a total of 80 individuals. Remarkably and similar to our study, they also found that all of the variants were located in exon 2, and a high percentage of the SNVs were localized to codon 44 (110/225; 49%). *MED12* variants were not limited to Finnish women, as 14/28 (50%) leiomyomas from eighteen black/colored South African women carried *MED12* variants [25]. These results are in agreement with our findings that 78% of black American women carried *MED12* variants. A recent study reported that rearrangements involving the 12q14∼15 region, and presumably associated with disruption of the *HMGA2* gene, are not associated with *MED12* mutations [26]. Among our cases, only two leiomyomas exhibited 12q14∼15 rearrangements, and neither of these tumors harbored *MED12* variants. Therefore, our sample size was too small to support or refute a hypothesis that rearrangements involving 12q14∼15 represent a separate genetic pathway.

Our study on outbred American women, including both white and black individuals, further extends the association of *MED12* variants and leiomyomas in various ethnic and racial groups. Further studies are needed to determine whether this association is causative or a reflection of other underlying variants in the myometrial genome which increase susceptibility to leiomyoma development.

## Supporting Information

Table S1 **Karyotype and demographic data for genotyped uterine leiomyoma.** A single tumor (leiomyoma) was genotyped from 148 patients. We have provided the karyotype of the genotyped tumor, size of the genotyped tumor, number of tumors detected (one/multiple), race, age at treatment, and *MED12* status of the genotyped tumor for each patient, where available. **^a^** Race is denoted by B for black American and W for white American. **^b^** *MED12* status is indicated by the specific mutation detected or WT, which denotes wild-type.
(XLS)
